# Assessing the impact of comorbidities on disease severity in COVID-19 patients requires consideration of age

**DOI:** 10.1097/MD.0000000000041360

**Published:** 2025-01-31

**Authors:** Aili Wang, Kun Li, Hui Sun, Yuan Wang, Huaie Liu

**Affiliations:** a Department of Infectious Diseases, The First Affiliated Hospital, Kunming Medical University, Kunming, Yunnan Province, China; b Department of General Surgery, Research Center of Digestive Diseases, Zhongnan Hospital, Wuhan University, Wuhan, Hubei Province, China.

**Keywords:** comorbidities, COVID-19, disease severity, older age, risk factor

## Abstract

Older age and comorbidities are risk factors for increased coronavirus disease 2019 (COVID-19) severity, but few studies have explored their interaction. This study aimed to assess the actual impacts of these factors on disease severity in COVID-19. The enrolled COVID-19 patients were divided into 4 age subgroups (≤44, 45–59, 60–74, and ≥75 years). Logistic regression analysis was conducted to determine the association between comorbidities and disease severity; Kappa consistency test was implemented to verify the study results. Of the 1663 patients with COVID-19, 287 had severe disease. The disease severity was correlated with the age-adjusted Charlson Comorbidity Index in each age group. In the 4 subgroups, the odds ratio of age-adjusted Charlson Comorbidity Index declined with age. After removing age interference, diabetes and cardio-cerebrovascular diseases were the main risk factors for severe disease in patients aged <75 years, whereas only chronic lung disease was associated with disease severity in patients aged ≥75 years. When comorbidities alone were used to predict disease severity, only the predictions were consistent with real outcomes in patients aged ≥75 years, compared with the predictions of high-risk comorbidities mentioned in World Health Organization and Chinese guidelines (Kappa 0.106, *P* < .05). Although older age and comorbidities were risk factors for severe COVID-19, their effects on disease severity varied across age groups. Additionally, comorbidities had a greater impact on COVID-19 severity in younger patients.

## Graphical abstract

**Figure:**
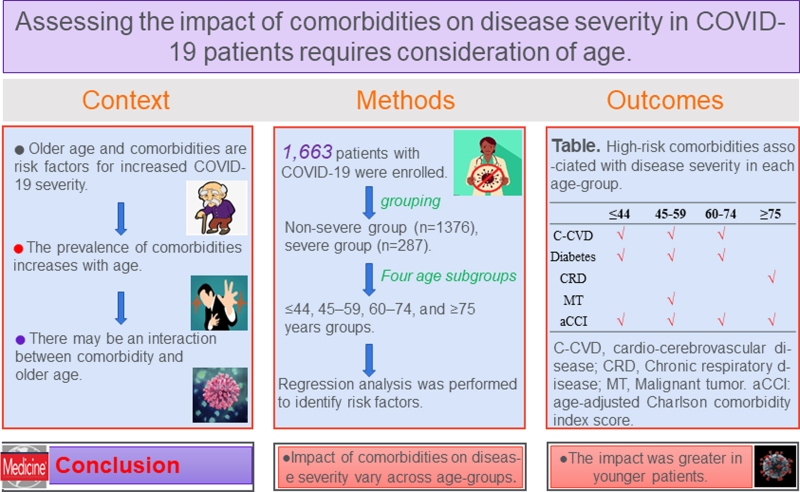

## 1. Introduction

The ongoing coronavirus disease 2019 (COVID-19) pandemic continues to pose significant challenges to global public health systems. As of April 28, 2024, over 775 million confirmed cases and more than 7 million deaths have been reported globally. Although the number of new cases and deaths has significantly decreased compared to before, it remains a major factor contributing to global public health issues. During the period from December 2023 to January 2024, there was an overall increase of 40% in new hospitalizations and 13% in admissions to intensive care units, and these populations experience the highest rates of mortality.^[1,2]^ Dealing with concentrated outbreaks requires the effective use of limited medical resources and increased success rates of treatment for COVID-19 patients.^[3]^ Therefore, clinicians should have a sufficient understanding of the risk factors associated with disease severity in COVID-19 patients. This knowledge can help in the early identification of patients who may become severely ill after infection with novel coronaviruses.

Many risk factors have been identified in the progression of COVID-19 into a severe and critical stage, including older age, male gender, underlying comorbidities such as hypertension, diabetes, chronic lung diseases, heart, liver and kidney diseases, and tumors.^[4–9]^ Laboratory parameters to monitor disease progression include lymphocytes, IL-6, C-reactive protein, ferritin, and D-dimer.^[10,11]^ Of these, older age and comorbidities are common risk factors for severe disease in COVID-19 patients.^[12–15]^ In elderly populations (defined by the World Health Organization [WHO] as patients aged ≥ 60 years),^[16]^ more than half of patients with COVID-19 have at least 1 comorbidity.^[14,17]^ The types and number of comorbidities vary in different age groups; the older the age, the higher the risk and types of comorbidities. It is important to ascertain whether older age and comorbidities are independent risk factors for severe disease in COVID-19 patients, or if this is a false impression created by the interaction between the 2 factors. This information can help identify patients that at a higher risk of developing severe illnesses and can be used to formulate policies aimed at preventing epidemics. Additionally, an accurate understanding of these risk factors is essential for the adjustment of disease treatment strategies. The goal of this study was to examine the impact of comorbidities on disease progression to the severe stage among COVID-19 patients after controlling for age.

## 2. Methods

### 2.1. Study population and data collection

This study was approved by the Research Ethics Commission of the Zhongnan Hospital of Wuhan University (No. 2020074). The requirement for informed consent was waived because the nature of this study was retrospective. In this retrospective cohort study, we enrolled 1663 hospitalized patients who tested positive for novel coronavirus nucleic acid between February 8 and March 16, 2020, in 3 clinical centers in Hubei Province, China. For all patients, we recorded the demographic (age and sex) and clinical data. Two experienced physicians reviewed each patient’s in-hospital case information and data regarding their previous history (information provided at admission), based on which all existing comorbidities were recorded. We followed the relevant guidelines of domestic^[18]^ and international^[19]^ agencies and outlined the common comorbidities of severe or critical patients for further research. The comorbidities included diabetes, chronic lung disease, chronic liver disease, chronic kidney disease, tumor, hematologic diseases, Alzheimer disease, and other cardiovascular and cerebrovascular diseases such as hypertension, coronary heart disease, cerebral hemorrhage, and cerebral infarction. For each patient, we also calculated the age-adjusted Charlson comorbidity index (aCCI) based on the Charlson comorbidity index rating scale.^[20]^

### 2.2. Clinical typing and grouping standards

We followed the clinical typing criteria of the Novel Coronavirus Pneumonia Treatment Protocol (Version 2)^[18]^ that was current at the time of patient admission. Patients were classified into 4 disease categories: light, normal, severe, and critical, based on clinical data such as the respiratory rate, oxygen saturation, blood gas levels, imaging results, mode of respiratory and circulatory support, and whether they were admitted to the ICU. Individuals in the light and normal categories were considered the non-severe group, and those in the severe and critical categories were considered the severe group. Based on the WHO criteria,^[16]^ we also divided the patients into elderly (≥60 years) and non-elderly (<60 years) groups and 4 age subgroups: young (aged ≤ 44 years; 370 cases), middle-aged (45–59 years; 494 cases), young olderly (60–74 years; 598 cases), and senior (≥75 years; 201 cases).

### 2.3. Statistical analysis

The SPSS statistical software (version 26.0; IBM Corp., USA) was used for statistical analysis of the data. Age was a normally distributed continuous variable expressed as the mean and standard deviation; inter-group differences were estimated with an independent *t* test. The aCCI was a non-normally distributed variable expressed as the median and interquartile range, and these values were compared with a Mann–Whitney *U* test. Sex, age groups, and comorbidities were categorical variables that were described as the number and percentage of cases and were compared with Fisher exact test. Logistic regression analysis was used to assess the impact of each comorbidity on disease severity in COVID-19 patients in different age groups. Variables that were associated with specific comorbidities (one comorbidity and aCCI) were excluded from the multiple logistic regression (MLR) analysis to avoid collinearity. The Kappa test was used to evaluate how consistently the comorbidities mentioned in this study or in the WHO and Chinese national guidelines predicted disease severity among age-stratified patients compared with the real clinical outcomes. Additionally, the presence of any high-risk comorbidities extracted from above indicated that the disease could progress to the severe or critical stages. Statistical significance was indicated by *P* < .05.

## 3. Results

### 3.1. Demographics and comorbidities

This study included 1663 patients with confirmed COVID-19. Of these, 287 (17.3%) were severe patients and 797 (47.9%) were male. Ages ranged from 9 to 97 years, with a mean of 57.0 ± 15.5 years. Patients in the severe group (66.8 ± 13.5 years) were significantly older than those in the non-severe group (55.0 ± 15.1 years) (Table 1). In the severe group, 214 (74.6%) patients were aged ≥ 60 years; the older the age, the higher the proportion of severe disease (Fig. 1).

**Table 1:** Demographic characteristics of enrolled patients with COVID-19.

Project	All patients (n = 1663)	Non-severe group (n = 1376)	Severe group (n = 287)	*P*
Sex (n, %)				.001
Male	797 (47.9)	634 (46.1)	163 (56.8)	
Female	866 (52.1)	742 (53.9)	124 (43.2)	
Age (years)	57.0 ± 15.5	55.0 ± 15.1	66.8 ± 13.5	<.001
Age groups (n, %)				<.001
<60 years	864 (52.0)	791 (57.5)	73 (25.4)	
≥60 years	799 (48.0)	585 (42.5)	214 (74.6)	
Age groups (n, %)				
≤44	370 (22.2)	351 (25.5)	19 (6.6)	
45–59	494 (29.7)	440 (32.0)	54 (18.8)	.003*
60–74	598 (36.0)	475 (34.5)	123 (42.9)	<.001*
≥75	201 (12.1)	110 (8.0)	91 (31.7)	<.001*

COVID-19 = coronavirus disease 2019.

* Compared to patients aged ≤ 44 years.

**Figure:**
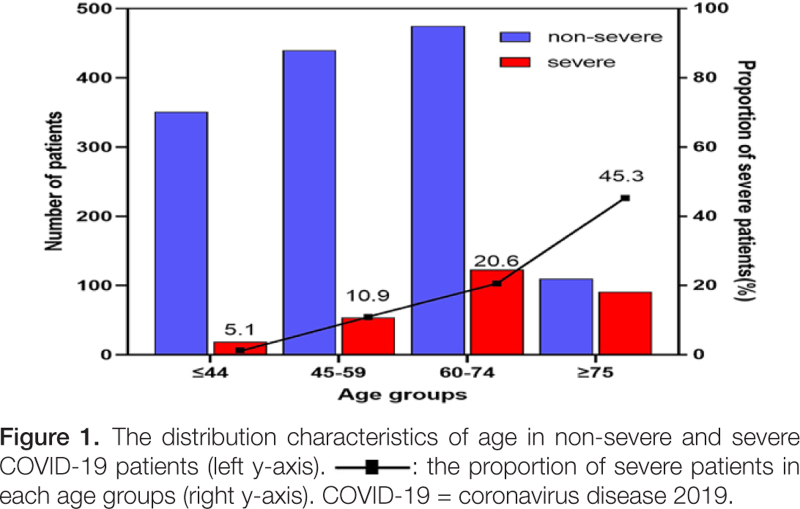


**Figure 2.**
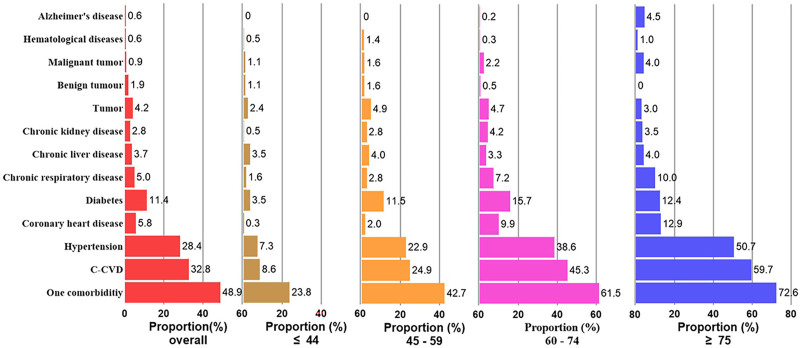
The distribution characteristics of comorbidities in different age groups. C-CVD = cardio-cerebrovascular diseases.

The prevalence of comorbidities varied across age groups. With increasing age, there was an increase in the proportion of patients with cardio-cerebrovascular disease, hypertension, coronary heart disease, chronic lung disease, malignant tumors, and Alzheimer disease. Among individuals aged ≥ 75 years, 72.6% had at least 1 comorbidity (Fig. 2). Overall, 48.9% of patients had 1 or more comorbidities. The most common comorbidity was cardio-cerebrovascular diseases (34.9%), followed by hypertension (28.4%) and diabetes (11.2%). The prevalence of 1 comorbidity, cardio-cerebrovascular disease, diabetes, chronic lung disease, malignancy, and Alzheimer disease was significantly higher in the severe group than in the non-severe group (all *P* < .05; Table 2).

**Table 2:** Comorbidities and related parameters in patients with COVID-19.

Project	All patients (n = 1663)	Non-severe group (n = 1376)	Severe group (n = 287)	*P*
One comorbidity (n, %)	813 (48.9)	606 (44.0)	207 (72.1)	<.001
C-CVD (n, %)	581 (34.9)	408 (29.7)	173 (60.3)	<.001
Hypertension	473 (28.4)	344 (25.0)	129 (45.0)	<.001
Coronary heart disease	95 (5.7)	60 (4.4)	35 (12.2)	<.001
Diabetes (n, %)	186 (11.2)	132 (9.6)	54 (18.8)	<.001
Chronic respiratory disease (n, %)	83 (5.0)	56 (4.1)	27 (9.4)	<.001
Chronic liver disease (n, %)	61 (3.7)	47 (3.4)	14 (4.9)	.288
Chronic kidney disease (n, %)	47 (2.8)	35 (2.5)	12 (4.2)	.167
Tumor (n, %)	69 (4.2)	51 (3.7)	18 (6.3)	.052
Benign tumor	15 (0.9)	12 (0.9)	3 (1.1)	.733
Malignant tumor	32 (1.9)	20 (1.5)	12 (4.2)	.007
Hematological diseases (n, %)	9 (0.5)	6 (0.4)	3 (1.0)	.058
Alzheimer disease (n, %)	10 (0.6)	3 (0.2)	7 (2.4)	<.001
aCCI (median [IQR])	2 (0–3)	1 (0–3)	3 (2–4)	<.001

aCCI = age-adjusted Charlson comorbidity index score, C-CVD = cardio-cerebrovascular disease, COVID-19 = coronavirus disease 2019, IQR = interquartile range.

**Figure 3.**
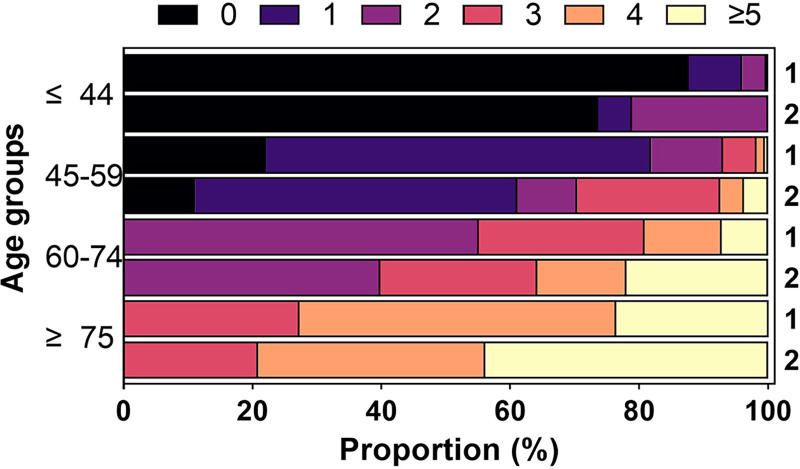
Proportion of different aCCI score in different clinical type by age groups. The stacked bar chart shows the relationship among aCCI score, age, and the disease severity. aCCI = age-adjusted Charlson comorbidity index score. (1) Non-severe group, (2) severe group.

**Figure 4.**
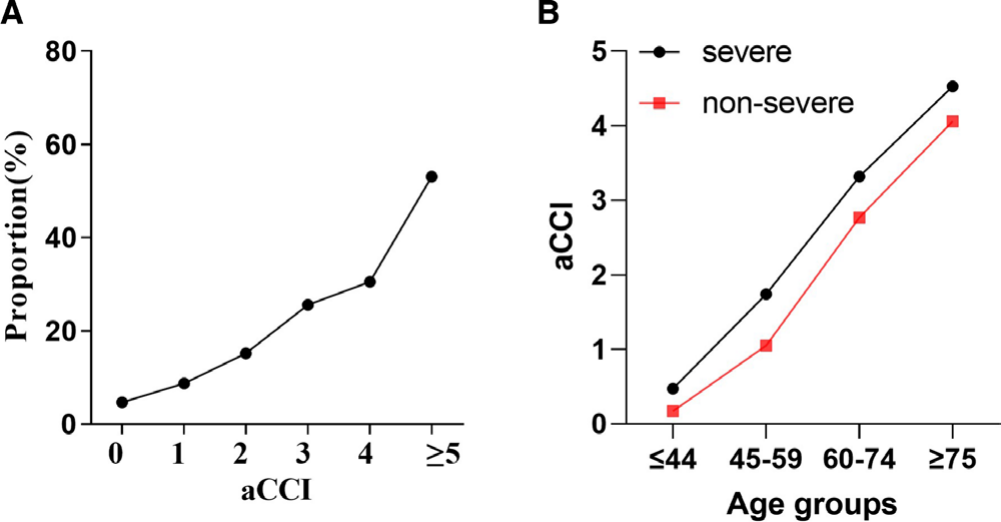
(A) Proportion of severe COVID-19 patients in different aCCI score. (B) aCCI average score in different clinical type across age groups. COVID-19 = coronavirus disease 2019.

The median aCCI in the severe group was 3 (interquartile range, 2–4), which is higher than that in the non-severe group (*P* < .001; Table 2). The proportion of patients with aCCI values of 0 or 1 declined with age; patients with low aCCI scores were more prevalent in the non-severe group. Among patients aged ≥ 45 years, the percentage of patients with aCCI scores ≥ 4 increased with age. No patients aged ≥ 75 years had aCCI values below 3, and there were more patients with high aCCI scores in the severe group (Fig. 3). Overall, aCCI scores were higher in the severe group than in the non-severe group. (Fig. 4A and B).

### 3.2. Risk factors by age groups

**Figure 5.**
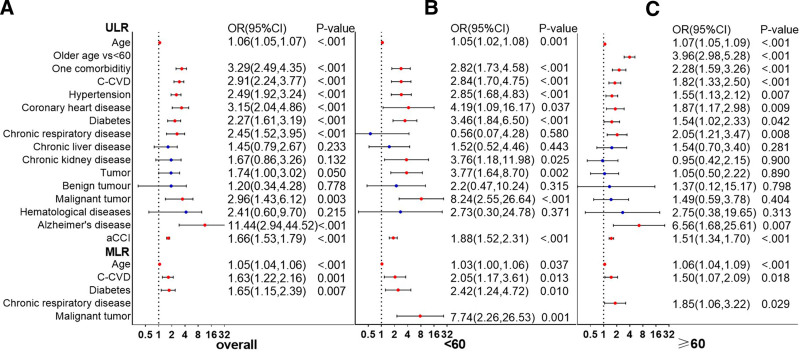
Impact assessment of comorbidities on disease severity of COVID-19 patients in overall, <60 and ≥60 years age groups. aCCI = age-adjusted Charlson comorbidity index score, C-CVD = cardio-cerebrovascular diseases, COVID-19 = coronavirus disease 2019, MLR = multivariate logistics regression analysis, ULR = univariate logistics regression analysis. *Note*: We did not include Alzheimer disease in the MLR analysis because of the low sample sizes. Blank spaces indicate that the data are not statistically significant.

Among all patients, the presence of 1 comorbidity, cardio-cerebrovascular disease, diabetes, chronic lung disease, malignancy, Alzheimer disease, and aCCI scores were associated with the severity of COVID-19 (*P* < .05). The results of the MLR analysis revealed that age (odds ratio [OR], 1.05; 95% confidence interval [95% CI], 1.04–1.06), diabetes (OR, 1.65; 95% CI, 1.14–2.39), and cardio-cerebrovascular disease (OR, 1.60; 95% CI, 1.20–2.13) were independent risk factors for severe disease (*P* < .05; Fig. 5).

One comorbidity, cardio-cerebrovascular disease, diabetes, and aCCI scores were thought to increase the risk of severe outcomes in patients aged <60 years or ≥60 years; however, the OR values was higher in the younger group. MLR analysis showed that age (OR, 1.03; 95% CI, 1.01–1.06), malignancy (OR, 7.55; 95% CI, 2.21–25.84), diabetes (OR, 2.16; 95% CI, 1.09–4.25), and cardio-cerebrovascular disease (OR, 1.94; 95% CI, 1.10–3.42) were independent risk factors for severe disease in patients aged < 60 years (*P* < .05). In addition, age (OR, 1.06; 95% CI, 1.04–1.09), chronic lung disease (OR, 1.88; 95% CI, 1.08–3.26), and cardio-cerebrovascular disease (OR, 1.43; 95% CI, 1.03–2.00) were independent risk factors for progression to severe disease among patients aged ≥ 60 years (*P* < .05; Fig. 5).

**Figure 6.**
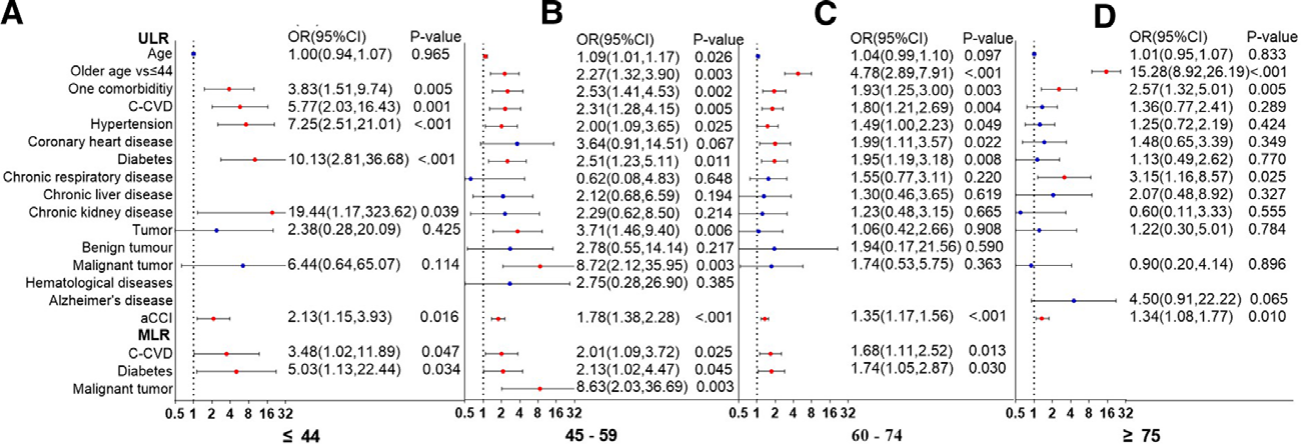
Impact assessment of comorbidities on disease severity in COVID-19 patients aged ≤44, 45 to 59, 60 to 74, and ≥75 years subgroups. *Note*: Blank spaces indicate that the data are not statistically significant. COVID-19 = coronavirus disease 2019.

Compared with patients aged ≤ 44 years, individuals aged 45 to 59, 60 to 74, and, ≥75 years had 2.27 (95% CI, 1.32–3.9), 4.78 (95% CI, 2.89–7.91), and 15.28 (95% CI, 8.92–26.19) times higher risk of severe outcomes, respectively. However, age was not an independent risk factor in any of the post-stratification age groups (Fig. 6). The following comorbidities predicted the risk of severe disease in each age group: diabetes (OR, 5.03; 95% CI, 1.13–22.44) and cardio-cerebrovascular disease (OR, 3.48; 95% CI, 1.02–11.89) in patients aged ≤ 44 years; diabetes (OR, 2.15; 95% CI, 1.03–4.47), cardio-cerebrovascular disease (OR, 1.85; 95% CI, 1.01–3.43), and malignancy (OR, 8.55; 95% CI, 2.02–36.20) in patients aged 45 to 59 years; diabetes (OR, 1.77; 95% CI, 1.07–2.94), and cardio-cerebrovascular disease (OR, 1.66; 95% CI, 1.10–2.49) in patients aged 60 to 74 years; and chronic lung disease (OR, 3.15; 95% CI, 1.16–8.57) in patients aged ≥75 years. Disease severity was linked to aCCI scores across all age groups, and the OR values decreased with age.

### 3.3. Comparison of different results

Compared with the WHO or Chinese national guidelines, our findings performed better in the age groups, ≤44 years and ≥75 years. Among patients aged ≥75 years, the comorbidities mentioned in the WHO or Chinese national guidelines cannot well predicted the risk of severe outcomes (*P* > .05), except in our findings (Kappa, 0.106; *P* < .05; Table 3).

**Table 3:** Consistency test of the predictions of comorbidities as risk factors in severe coronavirus disease 2019 (COVID-19) using different proposals.

High-risk comorbidities	≤44 years		45–59 years		60–74 years		≥75 years	
	Kappa	*P*	Kappa	*P*	Kappa	*P*	Kappa	*P*
Chinese guideline*	0.075	.076	0.137	<.001	0.088	.004	0.089	.174
WHO guideline†	0.117	.011	0.137	<.001	0.097	.002	0.087	.186
The study results	0.234	<.001	0.129	<.001	0.084	.011	0.106	.019

* Includes cardiovascular diseases (including hypertension), chronic lung diseases (chronic obstructive pulmonary disease, moderate to severe asthma), diabetes, chronic liver and kidney diseases, tumors, and immune deficiency diseases such as AIDS (National Health Commission of the People’s Republic of China, 2020).

† Includes diabetes, hypertension, heart disease, chronic lung disease, cerebrovascular disease, dementia, mental disorders, chronic kidney disease, immunosuppression, AIDS, and cancer (WHO, 2021).

## 4. Discussion

We found that age was an independent risk factor for severe disease among all patients. The probability of developing severe disease gradually increased with age. Compared with the younger age group (aged ≤ 44 years), patients aged ≥ 75 years were at 15.28 times higher relative risk of developing severe COVID-19. This is consistent with the findings of previous studies showing that older age is a significant risk factor for patients’ poor prognosis.^[21,22]^

In all COVID-19 patients, diabetes and cardio-cerebrovascular diseases were revealed as independent risk factors for disease severity. A meta-analysis^[23]^ revealed that the risk of severe disease was 1.79 and 3.92 times higher in COVID-19 patients with cardiovascular and cerebrovascular disease, respectively, than in patients without these comorbidities. Patients with diabetes have an increased risk of infection due to lowered immunity^[24]^; besides, they may also have a worse prognosis due to alveolar–capillary barrier dysfunction brought on by elevated angiotensin converting enzyme 2 expression in lung tissues.^[25,26]^ These findings suggest that diabetes and cardio-cerebrovascular disease may be indicators of severe disease in COVID-19 patients. However, some studies have reported that cardio-cerebrovascular disease (including hypertension, coronary heart disease), chronic lung disease, malignant tumors, and Alzheimer disease are age-related diseases.^[27,28]^ Therefore, it is unclear whether these comorbidities are directly linked to patients’ poor prognosis. Consequently, it is necessary to elucidate the actual risk posed by age and comorbidities separately in the development of severe disease in COVID-19 patients. This is particularly important in light of relevant studies (including the present study) showing that advanced age is a major risk factor for severe disease in COVID-19 patients.^[21,22]^

Univariate logistic regression analysis showed that age, 1 comorbidity, cardio-cerebrovascular disease, and diabetes were common risk factors for severe disease in patients aged <60 and ≥60 years. However, apart from age, all other risk factors posed a higher risk in younger patients aged <60 years, the MLR analysis revealed a similar pattern. Among the risk factors for severe disease in COVID-19 patients, there was a gradual decrease in the possibility of a single comorbidity leading to severe disease with increased age.

It was discovered that no single risk factor simultaneously affected the development of severe disease in all 4 age subgroups. According to the MLR analysis that without age as an independent risk factor, the ORs of risk factors (including diabetes and cardio-cerebrovascular disease) showed a decreasing trend with age in subgroups (except in the group aged ≥75 years). This also suggests that the impact of comorbidities on disease severity was stronger in younger age subgroups.

aCCI score was a risk factor for severe illness in the overall study population and in the variously stratified age groups. Moreover, the analysis of aCCI scores revealed a significant decrease in the impact of comorbidities on patients’ prognosis with advancing age. Several studies have reported that a high aCCI score can predict disease severity and mortality risk in individuals with COVID-19.^[29,30]^ Our results not only showed that high aCCI scores can predict poor prognosis in COVID-19 patients but also indicated that the impact of comorbidities on prognosis gradually diminished with age. This suggests that age should be taken into account when judging the prognosis of novel coronavirus infected patients based on their comorbidities, as there may be significant differences among patients in different age groups. Additionally, the impact of comorbidities was lower than expected in older patients. Previous studies^[21,31]^ have also indicated that the influence of the number of comorbidities on disease severity decreased with age. Comorbidities had a greater impact on disease severity in patients aged <50 years than in older patients.^[21]^ Therefore, clinicians need to discard the conventional notion that multiple comorbidities indicate a high risk of severe disease. This is especially important when taking protective measures for susceptible populations and allocating medical resources for patients with confirmed COVID-19.

We were also interested in the differences about how comorbidities affected disease severity in various age groups. MLR analysis revealed that, in patients aged <60 years, cardio-cerebrovascular disease, diabetes, and malignancy were the most significant comorbidities. In contrast, cardio-cerebrovascular disease and chronic lung disease were the most significant comorbidities in patients aged ≥60 years. The 4 age subgroups also showed variations in comorbidities that impacted COVID-19 patients in various ways in the different groups. Diabetes and cardio-cerebrovascular disease were independently linked to disease severity in patients in the younger age groups, but not in patients aged ≥75 years. Contrary to our expectations, we found that chronic pulmonary disease was the only comorbidity affecting disease severity in patients aged ≥ 75 years. Some studies^[5,17]^ have recognized cardio-cerebrovascular disease as a separate risk factor for severe disease. However, other studies have also determined that COVID-19 patients with hypertension do not exhibit a poor prognosis.^[27]^ The increased prevalence of hypertension in older patients may lead to the misinterpretation of these results, suggesting that advanced age is a primary risk factor. Our findings also confirmed this outcome.

The MLR analysis of the 4 age subgroups revealed that the ORs of cardio-cerebrovascular disease and diabetes decreased sequentially (or even lost statistical significance) without the interference of age. This suggests that clinicians should not blindly emphasize the control of cardio-cerebrovascular disease for COVID-19 patients in each age group. Some studies have shown that treatment with angiotensin converting enzyme inhibitors or angiotensin receptor blockers cannot significantly improve the clinical outcome or prognosis of COVID-19 patients.^[26]^ The present study shows that in patients aged ≥75 years, the control of chronic lung disease may be more meaningful for improving disease prognosis. This is because, for elderly patients with chronic lung disease, the COVID-19-induced destruction of the fragile respiratory system has more immediate consequences than the delayed deterioration process of the cardiovascular system.^[32]^

Even though the comorbidities analyzed here may be just 1 of many factors associated with the prognosis of patients with COVID-19, which may somewhat lower the generalizability of our results, we tested the reliability of our findings by evaluating the consistent effects of comorbidities on prognosis. The predictive results for our age-stratified groups were compatible with actual development to severe disease, and the prediction was especially effective for patients aged ≤44 and ≥75 years. In individuals aged ≥75 years, forecasts based on the high-risk comorbidities mentioned in either the current WHO or Chinese national guidelines were not consistent with the actual progression of severe disease. This suggests that a higher level of protection may be needed for advanced patients with chronic lung disease who have been diagnosed with COVID-19. In addition, these patients may require more advanced monitoring and preplanned treatment measures.

In previous studies on the relationship between comorbidities and disease severity in COVID-19 patients, the influence of age was often underestimated or ignored.^[33,34]^ Given the current general consensus that comorbidities and older age are both risk factors for severe disease in COVID-19 patients, it is difficult to truly distinguish their respective impact on patient prognosis. This would lead to confusion for clinicians to assess disease risk and select treatment options for patients at an early stage.

In this study, we found that although both older age and comorbidities were risk factors for severe disease in COVID-19 patients overall, the impact of comorbidities varied by patient age. On the one hand, aCCI showed a decreasing trend in all age groups of patients with COVID-19. This indicated that the effect of comorbidities on COVID-19 decreases rather than increases with age. This implied that younger patients should not be disregarded while assessing the disease severity with comorbidities.

On the other hand, the types of comorbidities that could lead to severe disease in patients with COVID-19 also varied by age. We found that diabetes and cardio -cerebrovascular diseases were high-risk factors for severe disease only in patients younger than 75 years of age. However, for patients aged ≥ 75 years, chronic lung disease was the only high-risk factor associated with severe disease among comorbidities. It was very important to accurately predict the prognosis of patients and reasonably allocate healthcare resources. Especially in the outbreak of the epidemic, the emergence of a large number of elderly patients, clinicians need to quickly triage the outpatient situation. The results of the Kappa test, which used the comorbidity method to predict patient severity, also suggested that the predictive method in this study was more reasonable for a population of ≥75 patients. In addition, our results partly explained the wide variation in the impact of cardio-cerebrovascular disease on COVID-19 prognosis in relevant clinical studies.^[27,28,35–37]^ It makes more sense to stratify the risk of comorbidities by age because there are obvious differences in how comorbidities affect disease in various age groups. Because the impact of comorbidities on severe disease in COVID-19 varied with age, the relevant studies had come to different conclusions without taking age into account.

There are some limitations to our study. First, in the initial stage of data collection, we gathered early-stage epidemic data of COVID-19 patients. Due to ongoing advancements in the clinical understanding of this disease, some contemporary diagnostic and treatment standards may differ from those used in the early stages of the epidemic. Second, this was a retrospective multicenter investigation, several comorbidities (such Alzheimer disease) occurred at low frequencies in the younger age groups. Due to the extremely low sample sizes, we did not perform any further analysis of these comorbidities.

## 5. Conclusions

While old age and comorbidities were risk factors for severe COVID-19, comorbidities were more of a threat for younger patients. There were significant differences in the impacts of comorbidities on individuals in various age groups. Thus, we should take age into account when assessing how various comorbidities affect the severity of the disease in COVID-19 patients. This is crucial for accurate evaluation of patient prognosis and rational allocation of medical resources in outbreak areas.

## Acknowledgments

We acknowledge the staff of Zhongnan Hospital of Wuhan University for their cooperation and Professors ZWL of department of Pathogen Biology and Immunology, and ZJ of department of Public Health, Kunming Medical University for their guidance on article statistics and graph production.

## Author contributions

**Conceptualization:** Aili Wang, Kun Li, Huaie Liu.

**Data curation:** Aili Wang, Hui Sun, Yuan Wang.

**Formal analysis:** Aili Wang, Hui Sun, Huaie Liu.

**Supervision:** Kun Li, Huaie Liu.

**Validation:** Huaie Liu.

**Writing – original draft:** Aili Wang.

**Writing – review & editing:** Aili Wang, Huaie Liu.
